# Supplementary material for: Thiorhodovibrio frisius and Trv. litoralis spp. nov., Two Novel Members from a Clade of Fastidious Purple Sulfur Bacteria That Exhibit Unique Red-Shifted Light-Harvesting Capabilities
Source: Microorganisms. 2023 Sep 25;11(10):2394. doi: 10.3390/microorganisms11102394 (PMC10609205; doi:10.3390/microorganisms11102394)
Supplement: Supplementary file 1 [file microorganisms-11-02394-s001.zip › Figure S1_2023.09.05.pptx]

## Slide 1
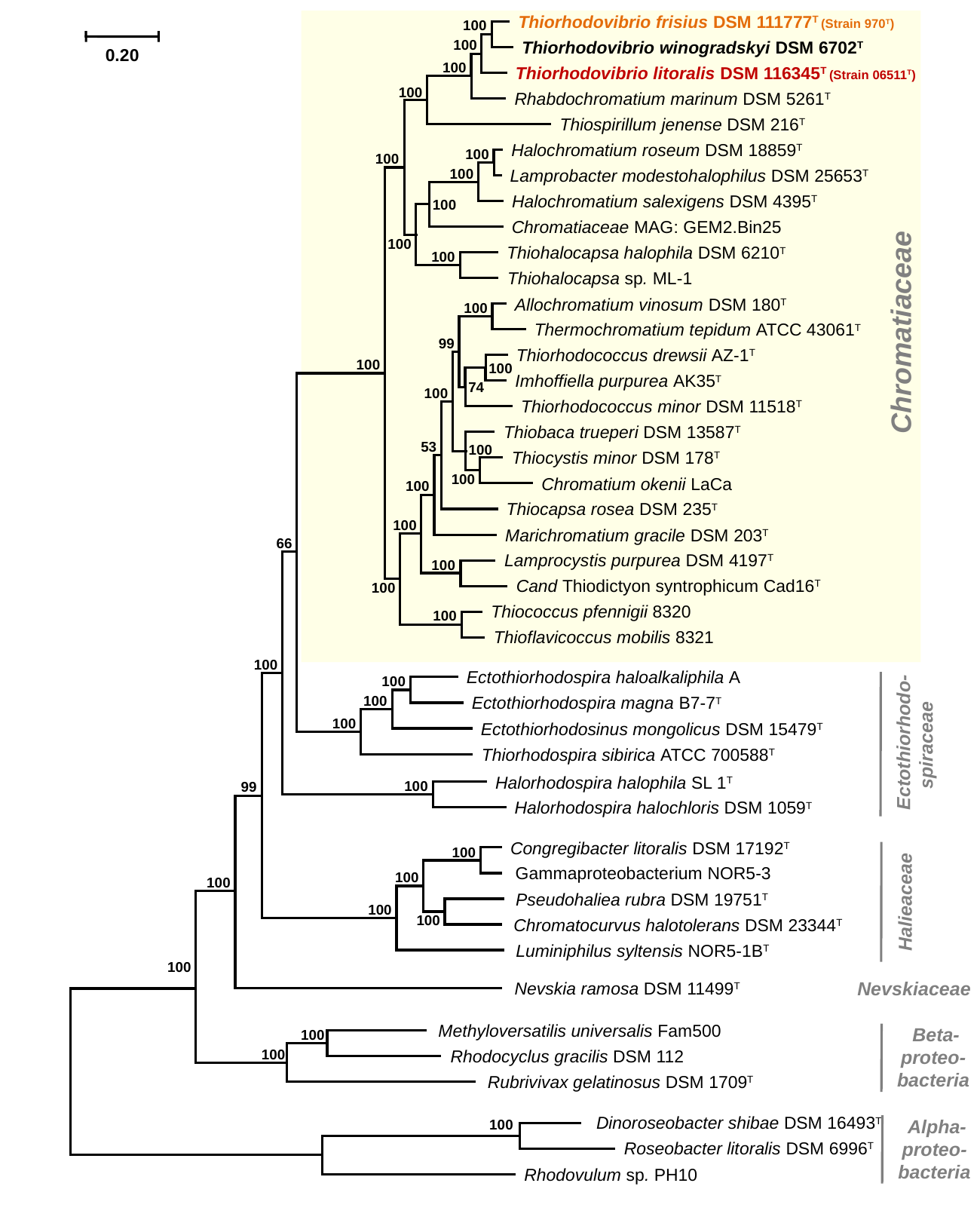

Thiorhodovibrio frisius DSM 111777T (Strain 970T)
 Thiorhodovibrio winogradskyi DSM 6702T
 Thiorhodovibrio litoralis DSM 116345T (Strain 06511T)
 Rhabdochromatium marinum DSM 5261T
 Thiospirillum jenense DSM 216T
 Halochromatium roseum DSM 18859T
 Lamprobacter modestohalophilus DSM 25653T
 Halochromatium salexigens DSM 4395T
 Chromatiaceae MAG: GEM2.Bin25
 Thiohalocapsa halophila DSM 6210T
 Thiohalocapsa sp. ML-1
 Allochromatium vinosum DSM 180T
 Thermochromatium tepidum ATCC 43061T
 Thiorhodococcus drewsii AZ-1T
 Imhoffiella purpurea AK35T
 Thiorhodococcus minor DSM 11518T
 Thiobaca trueperi DSM 13587T
 Thiocystis minor DSM 178T
 Chromatium okenii LaCa
 Thiocapsa rosea DSM 235T
 Marichromatium gracile DSM 203T
 Lamprocystis purpurea DSM 4197T
 Cand Thiodictyon syntrophicum Cad16T
 Thiococcus pfennigii 8320
 Thioflavicoccus mobilis 8321
100
100
100
100
100
100
100
100
100
100
100
99
100
100
74
100
53
100
100
100
100
100
100
100
0.20
 Chromatiaceae
66
100
100
100
100
100
 Ectothiorhodospira haloalkaliphila A
 Ectothiorhodospira magna B7-7T
 Ectothiorhodosinus mongolicus DSM 15479T
 Thiorhodospira sibirica ATCC 700588T
 Halorhodospira halophila SL 1T
 Halorhodospira halochloris DSM 1059T
100
100
100
 Ectothiorhodo-
spiraceae
100
99
100
100
100
100
 Congregibacter litoralis DSM 17192T
 Gammaproteobacterium NOR5-3
 Pseudohaliea rubra DSM 19751T
 Chromatocurvus halotolerans DSM 23344T
 Luminiphilus syltensis NOR5-1BT
 Halieaceae
 Nevskiaceae
 Nevskia ramosa DSM 11499T
 Methyloversatilis universalis Fam500
 Rhodocyclus gracilis DSM 112
 Rubrivivax gelatinosus DSM 1709T
 Beta-
proteo-
bacteria
 Dinoroseobacter shibae DSM 16493T
Roseobacter litoralis DSM 6996T
 Rhodovulum sp. PH10
 Alpha-
proteo-
bacteria
100
