# Supplementary material for: Thiorhodovibrio frisius and Trv. litoralis spp. nov., Two Novel Members from a Clade of Fastidious Purple Sulfur Bacteria That Exhibit Unique Red-Shifted Light-Harvesting Capabilities
Source: Microorganisms. 2023 Sep 25;11(10):2394. doi: 10.3390/microorganisms11102394 (PMC10609205; doi:10.3390/microorganisms11102394)
Supplement: Supplementary file 1 [file microorganisms-11-02394-s001.zip › Figure S2_2023.09.05.pdf]

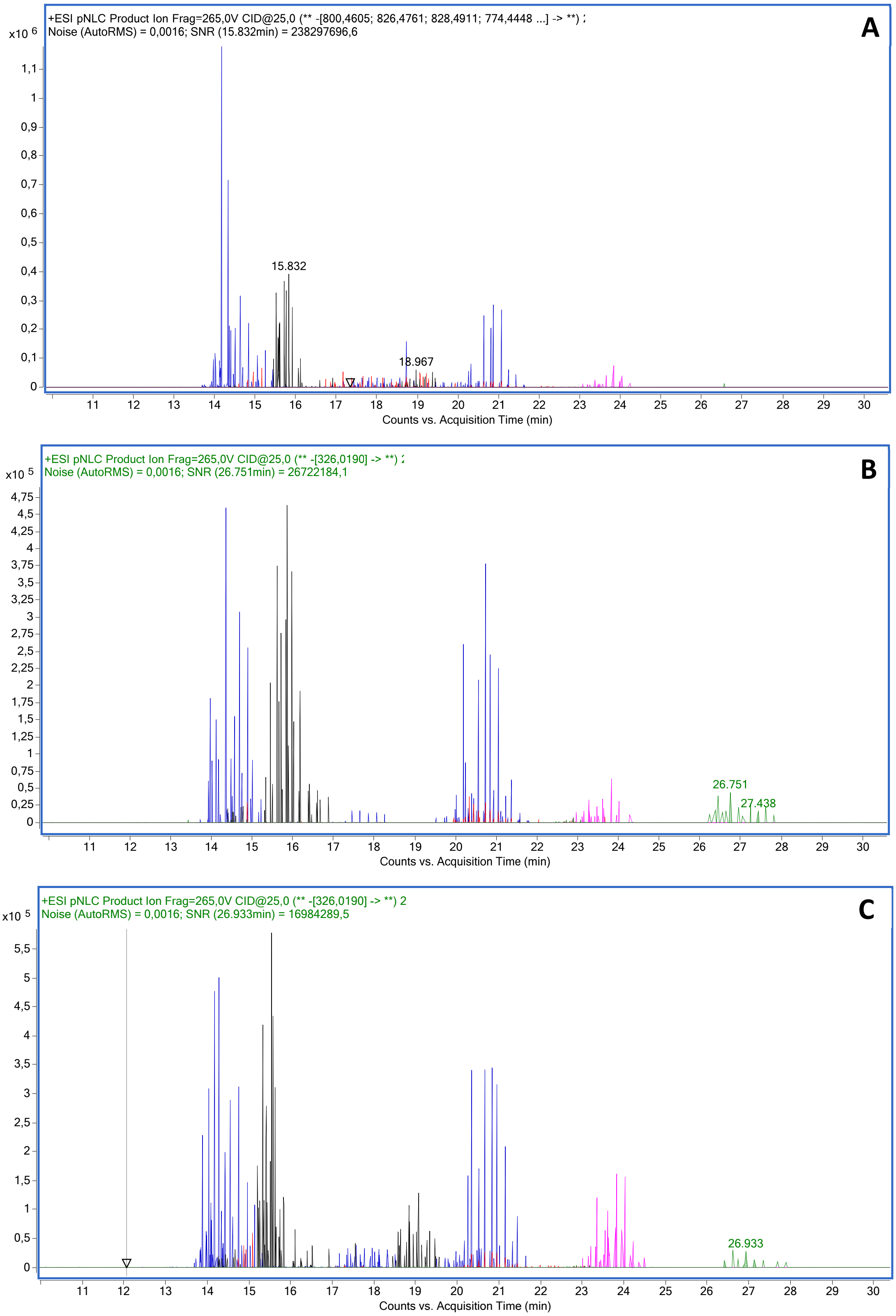

Figure S2. Neutral loss spectra of intact polar lipids found in (**A**) *Trv. frisius* DSM 111777^{T}, (**B**) *Trv. winogradskyi* DSM 6702^{T} and (**C**) *Trv. litoralis* strain 06511^{T}. Blue: Neutral loss of m/z 141.0191corresponding to phosphatidylethanolamine (~ 14 min) and lyso-phosphatidylethanolamine (~ 21 min); Red: Neutral loss of m/z 172.0137 corresponding to phosphatidylglycerol (~ 15 min) and lysophosphatidylglycerol (~ 20 min); Black: Neutral loss of one corelipid and phosphate, corresponding to diphosphatidylglycerol (~ 16 min). Pink: Neutral loss of m/z 270.06 (~ 23 min) corresponding to unknown glycolipids. Green: Neutral loss of m/z 326.02 corresponding to unknown glycolipids (~26 min).
