# Supplementary material for: Thiorhodovibrio frisius and Trv. litoralis spp. nov., Two Novel Members from a Clade of Fastidious Purple Sulfur Bacteria That Exhibit Unique Red-Shifted Light-Harvesting Capabilities
Source: Microorganisms. 2023 Sep 25;11(10):2394. doi: 10.3390/microorganisms11102394 (PMC10609205; doi:10.3390/microorganisms11102394)
Supplement: Supplementary file 1 [file microorganisms-11-02394-s001.zip › Figure S3_2023.09.20.pdf]

A) 16S rRNA gene

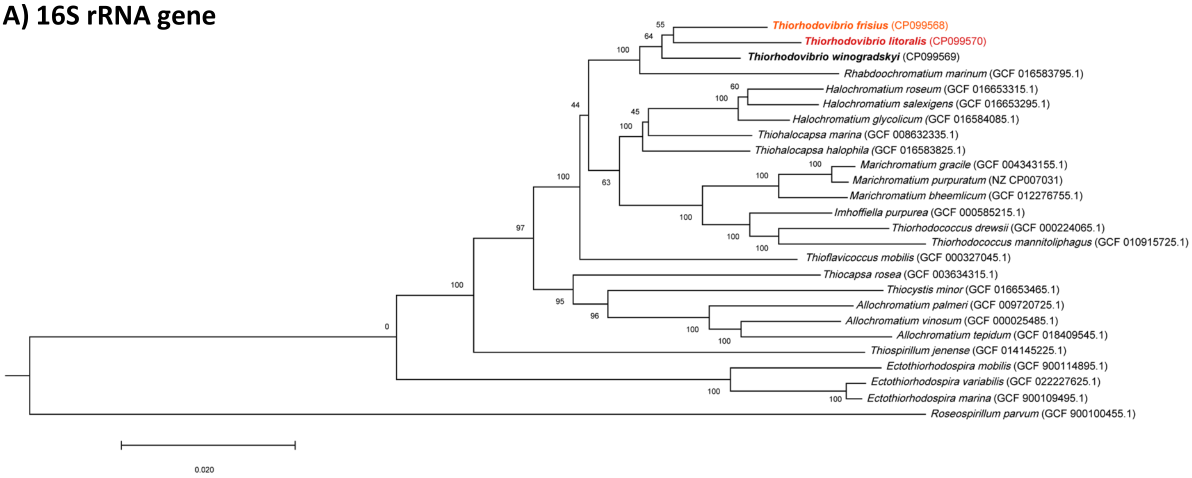

B) Genome

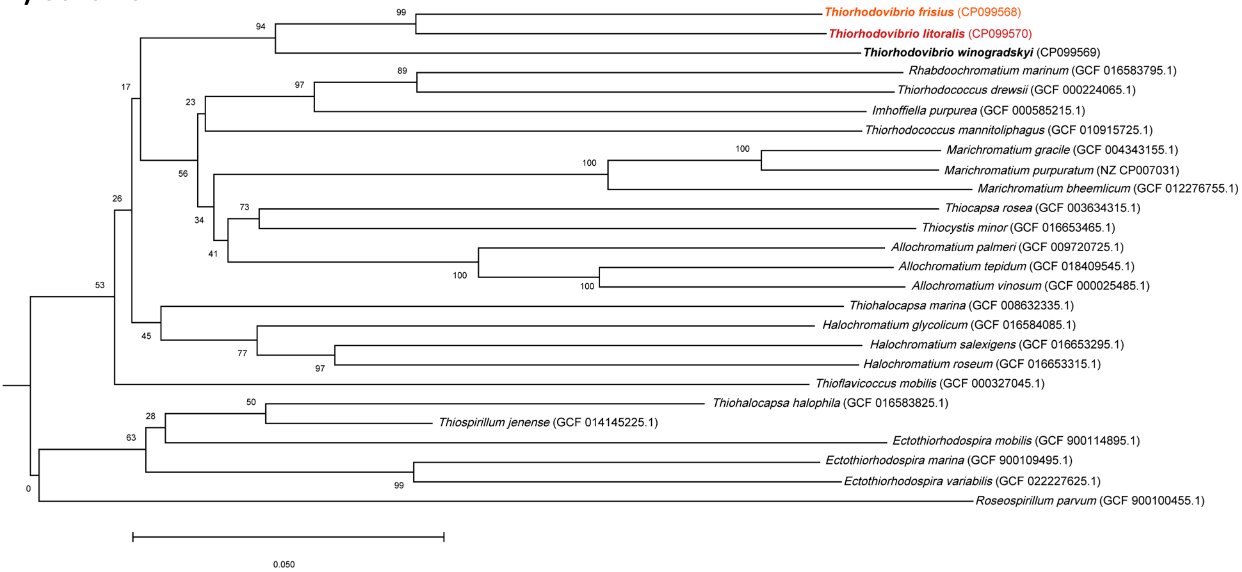

C) Proteome

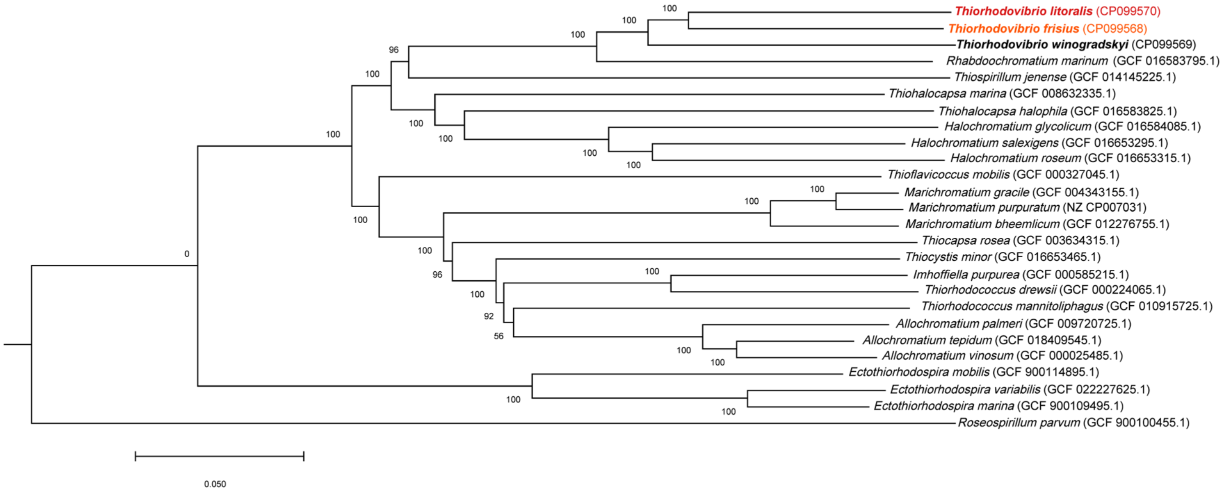

Figure S3. TYGS based phylogenetic trees. Trees rooted at midpoint were inferred with FastME 2.1.6.1 from GBDP distances calculated with formula d_5 using **(A)** 16S rRNA gene, **(B)** genome, or **(C)** whole-proteome amino acid sequences. Pseudo-bootstrap support values >60% from 100 replications are depicted, with an average branch support of 87.8, 67.9, and 97.4% respectively.
