# Supplementary material for: Thiorhodovibrio frisius and Trv. litoralis spp. nov., Two Novel Members from a Clade of Fastidious Purple Sulfur Bacteria That Exhibit Unique Red-Shifted Light-Harvesting Capabilities
Source: Microorganisms. 2023 Sep 25;11(10):2394. doi: 10.3390/microorganisms11102394 (PMC10609205; doi:10.3390/microorganisms11102394)
Supplement: Supplementary file 1 [file microorganisms-11-02394-s001.zip › Table S2_2023.09.05.pdf]

		16S rRNA gene (%)					dDDH (d_4, %)					ANI (%)				
		1	2	3	4	5	1	2	3	4	5	1	2	3	4	5
Trv. frisius	1	-	98.0	97.5	96.2	92.5	-	26.5	34.2	23.5	27.0	-	81.9	83.7	77.6	68.8
Trv. winogradskyi	2		-	97.5	96.6	92.2		-	25.3	22.7	29.0		-	79.1	77.7	68.9
Trv. litoralis	3			-	95.5	92.6			-	24.0	27.8			-	76.9	68.9
Rhabdochromatium marinum	4				-	93.0				-	21.7				-	68.9
Thiospirillum jenense	5					-					-					-

Table S2. Genetic speciation characteristics of *Thiorhodovibrio sp.* and associated photosynthetic relatives. A Comparison of three genome-sequenced *Thiorhodovibrio* strains, *Rhabdochromatium marinum* DSM 5261^T, and *Thiospirillum jenense* DSM 216^T using 16S rRNA gene similarity, Digital DNA-DNA hybridization values (dDDH) and Average Nucleotide Identity (ANI) values between the five genomes.
