# Supplementary material for: Thiorhodovibrio frisius and Trv. litoralis spp. nov., Two Novel Members from a Clade of Fastidious Purple Sulfur Bacteria That Exhibit Unique Red-Shifted Light-Harvesting Capabilities
Source: Microorganisms. 2023 Sep 25;11(10):2394. doi: 10.3390/microorganisms11102394 (PMC10609205; doi:10.3390/microorganisms11102394)
Supplement: Supplementary file 1 [file microorganisms-11-02394-s001.zip › Tables S3-S5_2023.09.05.pdf]

Table S3. Genes of *Thiorhodovibrio* spp. involved in osmotic adaptation.

"Glycine" related genes	Description	*Trv. frisius* 006511^T	*Trv. winogradskyi* DSM 6702^T	*Trv. litoralis* stain 970^T
betL	Glycine betaine-Na(+) symporter	-	-	Thiosp_03236
gbuA	Glycine betaine/carnitine transport ATP-binding	-	-	Thiosp_01381
gbuB	Glycine betaine/carnitine transport permease	-	Thiowin_03044	Thiosp_04329
gbuC 1	Glycine betaine/carnitine transport binding precursor	Thiofri_04750	Thiowin_03045	Thiosp_04328
gbuC 2	Glycine betaine/carnitine transport binding precursor	-	Thiowin_03937	-
gcvH	Glycine cleavage system H protein	Thiofri_02064	Thiowin_03799	Thiosp_02802
gcvPA	putative glycine dehydrogenase [decarboxylating]subunit 1	Thiofri_02063	Thiowin_03800	Thiosp_02803
gcvPB	putative glycine dehydrogenase [decarboxylating]subunit 2	Thiofri_02062	Thiowin_03801	Thiosp_02804
G/S N-methyltransferase	Glycine/sarcosine N-methyltransferase	Thiofri_00397	Thiowin_05092	Thiosp_00206
G/S/D N-methyltransferase	Glycine/sarcosine/dimethylglycine N-methyltransferase	-	Thiowin_02708	-
glyQ	Glycine--tRNA ligase alpha subunit	Thiofri_04609	Thiowin_04789	Thiosp_00297
glyS	Glycine--tRNA ligase beta subunit	Thiofri_04610	Thiowin_04790	Thiosp_00298
opuAB	Glycine betaine transport system permease	Thiofri_04749	-	-
opuCA	Glycine betaine/carnitine/choline transport ATP-binding	-	Thiowin_01936	Thiosp_01005
opuCB	Glycine betaine/carnitine/choline transport system permease	-	Thiowin_01935	Thiosp_01004
opuD 1	Glycine betaine transporter	Thiofri_02247	Thiowin_01524	Thiosp_03115
opuD 2	Glycine betaine transporter	-	Thiowin_03936	Thiosp_03560
proV	Glycine betaine/L-proline transport ATP-binding	Thiofri_04748	Thiowin_03043	Thiosp_04330
purD	Phosphoribosylamine--glycine ligase	Thiofri_03608	Thiowin_01621	Thiosp_01166
N-methyltransferase	Sarcosine/dimethylglycine N-methyltransferase	Thiofri_00398	Thiowin_05093	Thiosp_00205
thiO	Glycine oxidase	Thiofri_00930	Thiowin_04410	
**"Sodium (Na+)" related genes**				
Na+ SNF	Na+-dependent transporters of the SNF family protein	Thiofri_01513	Thiowin_03606	Thiosp_02360
NhaD 1	Na+/H+ antiporter, NhaD family	Thiofri_01606	Thiowin_02804	Thiosp_02827
NhaD 2	Na+/H+ antiporter, NhaD family	-	-	Thiosp_02340
DASS 1	transporter, divalent anion:Na+ symporter (DASS)family	Thiofri_01961	Thiowin_01270	Thiosp_02339
DASS 2	transporter, divalent anion:Na+ symporter (DASS)family	Thiofri_02589	Thiowin_01655	Thiosp_01897
mrpF 1	Sodium-cholate efflux protein MrpF	Thiofri_02888	Thiowin_03711	Thiosp_01848
mrpF 2	Sodium-cholate efflux protein MrpF	-	-	Thiosp_03149
nhaK	Sodium, potassium, lithium and rubidium/H(+) antiporter	Thiofri_04950	Thiowin_01384	-
ntpD	V-type sodium pump subunit D	-	-	Thiosp_00061
SSS small	putative solute:sodium symporter small subunit	Thiofri_02774	Thiowin_00883	Thiosp_02403
sodium/calcium exchanger 1	sodium/calcium exchanger 1	-	Thiowin_03917	-
SSS	transporter, solute:sodium symporter (SSS) family	-	Thiowin_03497	-

Table S4. Genes of *Trv. frisius* DSM111777^T, *Trv. winogradskyi* DSM 6702^T, *Trv. litoralis* 06511^T potentially involved in the detoxification of oxygen and oxygen radicals.

"Oxidase/ Peroxidase" related genes	**Description**	***Trv. frisius 006511^T***	***Trv. winogradskyi DSM 6702^T***	***Trv. litoralis stain 970^T***
bsaA	Glutathione peroxidase	Thiofri_01328	Thiowin_03142	Thiosp_01211
cbb3 subunit II	cbb3-type cytochrome c oxidase subunit II	Thiofri_01951	Thiowin_01259	Thiosp_02327
ccpA	Cytochrome c551 peroxidase precursor	Thiofri_04511	-	Thiosp_04559
coproporphyrinogen III oxidase	coproporphyrinogen III oxidase	Thiofri_04847	Thiowin_00940	Thiosp_03571
coproporphyrinogen III oxidase	coproporphyrinogen III oxidase	Thiofri_02232	-	-
coproporphyrinogen III oxidase	coproporphyrinogen III oxidase	Thiofri_04860	-	-
cpo	Non-heme chloroperoxidase	Thiofri_03789	Thiowin_01227	Thiosp_01564
cydA	Cytochrome d ubiquinol oxidase subunit 1	Thiofri_01486	Thiowin_01374	Thiosp_02771
cydB	Cytochrome d ubiquinol oxidase subunit 2	Thiofri_01485	Thiowin_01373	Thiosp_02772
CcoG	cytochrome c oxidase accessory protein CcoG	Thiofri_01954	Thiowin_01262	Thiosp_02330
cytC oxidase, cbb3-type, subunit III	cytochrome c oxidase, cbb3-type, subunit III	Thiofri_01906	Thiowin_04603	Thiosp_02334
Cyt oxidase complex assembly protein 1	Cytochrome oxidase complex assembly protein 1	Thiofri_02898	-	-
Cyt oxidase maturation protein, cbb3-type	cytochrome oxidase maturation protein, cbb3-type	Thiofri_01957	Thiowin_01265	-
fixN	Cytochrome c oxidase subunit 1	Thiofri_01950	Thiowin_01258	Thiosp_02326
fixP	Cytochrome c oxidase subunit III	Thiofri_01953	Thiowin_01261	Thiosp_02329
garA	Glutathione amide-dependent peroxidase	Thiofri_01026	Thiowin_04194	Thiosp_01050
hemF	Coproporphyrinogen-III oxidase, aerobic	Thiofri_02877	Thiowin_03700	Thiosp_01856
hemN 1	Oxygen-independent coproporphyrinogen-III oxidase 1	Thiofri_02216	Thiowin_00485	Thiosp_00537
hemN 2	Oxygen-independent coproporphyrinogen-III oxidase	Thiofri_04147	Thiowin_00568	Thiosp_00723
hemN 3	Oxygen-independent coproporphyrinogen-III oxidase	Thiofri_04572	Thiowin_00766	Thiosp_01551
hemY	Protoporphyrinogen oxidase	Thiofri_01523	-	Thiosp_02467
katG 1	Catalase-peroxidase	-	Thiowin_01079	Thiosp_03123
katG 2	Catalase-peroxidase	-		Thiosp_03157
katG 3	Catalase-peroxidase	-		Thiosp_03162
livQ 1	6'''-hydroxyparomomycin C oxidase	Thiofri_03586	Thiowin_02493	-
livQ 2	6'''-hydroxyparomomycin C oxidase	-	Thiowin_04316	-
nadB	L-aspartate oxidase	Thiofri_02565	Thiowin_02216	Thiosp_02585
neoG	Paromamine 6'-oxidase	Thiofri_02821	-	-
nox	NADH oxidase	Thiofri_04519	Thiowin_04469	Thiosp_04602
pdxH	Pyridoxine/pyridoxamine 5'-phosphate oxidase	Thiofri_01096	Thiowin_04023	Thiosp_03618
protoporphyrinogen oxidase 1	protoporphyrinogen oxidase	Thiofri_02110	Thiowin_04300	Thiosp_01920
protoporphyrinogen oxidase 2	protoporphyrinogen oxidase	-	Thiowin_03758	-
protoporphyrinogen oxidase 3	protoporphyrinogen oxidase	-	Thiowin_03595	-
sodB 1	Superoxide dismutase [Fe]	Thiofri_01934	Thiowin_02877	Thiosp_02682
sodB 2	Superoxide dismutase [Fe]	-	Thiowin_03340	Thiosp_03213
sodC 1	Superoxide dismutase [Cu-Zn] precursor	-	Thiowin_03453	Thiosp_03187
sodC 2	Superoxide dismutase [Cu-Zn] precursor	-	Thiowin_03557	-
thiO	Glycine oxidase	Thiofri_00930	Thiowin_04410	-
wbpB 1	UDP-N-acetyl-2-amino-2-deoxy-D-glucuronate oxidase	-	-	Thiosp_00142
wbpB 2	UDP-N-acetyl-2-amino-2-deoxy-D-glucuronate oxidase	-	-	Thiosp_04203

Table S5. Motility related genes annotated through KEGG online database for *Trv. frisius* DSM111777^{T}, *Trv. winogradskyi* DSM 6702^{T}, *Trv. litoralis* 06511^{T}.

Gene	Description	KO code	*Trv. frisius 006511^{T}*	*Trv. winogradskyi DSM 6702^{T}*	*Trv. litoralis stain 970^{T}*
flgA	flagellar basal body P-ring formation protein FlgA	K02386	Thiofri_04680	Thiowin_04913	Thiosp_00317
flgB	flagellar basal-body rod protein FlgB, proximal rod	K02387	Thiofri_04683	Thiowin_04910	Thiosp_00314
flgC	flagellar basal-body rod protein FlgC, proximal rod	K02388	Thiofri_04684	Thiowin_04909	Thiosp_00313
flgD	flagellar basal-body rod modification protein FlgD	K02389	Thiofri_04685	Thiowin_04908	Thiosp_00312
flgE	flagellar hook protein FlgE	K02390	Thiofri_04686	Thiowin_04907	Thiosp_00311
flgF	flagellar basal-body rod protein FlgF, proximal rod	K02391	Thiofri_04687	Thiowin_04906	Thiosp_00310
flgG	flagellar basal-body rod protein FlgG, distal rod	K02392	Thiofri_04688	Thiowin_04905	Thiosp_00309
flgH	flagellar L-ring protein FlgH	K02393	Thiofri_04689	Thiowin_04904	Thiosp_00308
flgI	flagellar P-ring protein FlgI	K02394	Thiofri_04690	Thiowin_04903	Thiosp_00307
flgJ	peptidoglycan hydrolase FlgJ	K02395	Thiofri_04691	Thiowin_04902	Thiosp_00306
flgK	flagellar hook-associated protein 1, hook-filament junction	K02396	Thiofri_04692	Thiowin_04901	Thiosp_00305
flgL	flagellar hook-associated protein 3 FlgL, hook-filament junction	K02397	Thiofri_04693	Thiowin_04900	Thiosp_00304
flgM	negative regulator of flagellin synthesis FlgM	K02398	**(Thiofri_04678)**	Thiowin_04914	Thiosp_00318
-	- unknown gene insertion -	-	**Thiofri_04679**	-	-
flgN	flagellar biosynthesis protein FlgN	K02399	Thiofri_04677	Thiowin_04915	Thiosp_00319
flhA	flagellar biosynthesis protein FlhA	K02400	Thiofri_04665	Thiowin_04927	Thiosp_00333
flhB	flagellar biosynthesis protein FlhB	K02401	Thiofri_04664	Thiowin_04928	Thiosp_00334
fliA, whiG	RNA polymerase sigma factor FliA	K02405	Thiofri_04668	Thiowin_04924	Thiosp_00330
fliC, hag	Flagellin, filament	K02406	Thiofri_01373, Thiofri_01400	Thiowin_03649	Thiosp_00043, Thiosp_01323, Thiosp_01326, Thiosp_01354
fliD	flagellar hook-associated protein 2, cap	K02407	Thiofri_01375	Thiowin_03647	Thiosp_01328
fliE	flagellar hook-basal body complex protein FliE, proximal rod	K02408	Thiofri_04467	Thiowin_04887	Thiosp_04408
fliF	flagellar M-ring protein FliF	K02409	Thiofri_04466	Thiowin_04886	Thiosp_04407
fliG	flagellar motor switch protein FliG, C-ring	K02410	Thiofri_04465	Thiowin_04885	Thiosp_04406
fliH	flagellar assembly protein FliH	K02411	Thiofri_04464	Thiowin_04884	Thiosp_04405
fliI	flagellum-specific ATP synthase	K02412	Thiofri_04463	Thiowin_04883	Thiosp_04404
fliJ	flagellar protein FliJ	K02413	Thiofri_04462	Thiowin_04882	Thiosp_04403
fliK	flagellar hook-length control protein FliK	K02414	Thiofri_04461	Thiowin_04881	Thiosp_04402
fliL	flagellar protein FliL	K02415	Thiofri_02262	-	Thiosp_03578
fliM	flagellar motor switch protein FliM, C-ring	K02416	Thiofri_04658	Thiowin_04934	Thiosp_00340
fliN	flagellar motor switch protein FliN, C-ring	K02417	Thiofri_04659	Thiowin_04933	Thiosp_00339
fliO, fliZ	flagellar protein FliO/FliZ	K02418	**(Thiofri_04660)**	Thiowin_04932	Thiosp_00338
fliP	flagellar biosynthesis protein FliP	K02419	Thiofri_04661	Thiowin_04931	Thiosp_00337
fliQ	flagellar biosynthesis protein FliQ	K02420	Thiofri_04662	Thiowin_04930	Thiosp_00336
fliR	flagellar biosynthesis protein FliR	K02421	Thiofri_04663	Thiowin_04929	Thiosp_00335
fliS	flagellar secretion chaperone FliS	K02422	Thiofri_01376	Thiowin_03644	Thiosp_01329
motA	chemotaxis protein MotA, stator	K02556	Thiofri_04378	Thiowin_00702	Thiosp_00325, Thiosp_04621
motB	chemotaxis protein MotB, stator	K02557	Thiofri_04377	Thiowin_00703	Thiosp_00324, Thiosp_04620
rpoD	RNA polymerase primary sigma factor	K03086	Thiofri_02358	Thiowin_02014	Thiosp_02643
rpoN	RNA polymerase sigma-54 factor	K03092	Thiofri_04727	Thiowin_00314	Thiosp_04350
flrA, fleQ, flaK	sigma-54 dependent transcriptional regulator, flagellar regulatory protein	K10941	Thiofri_00976, Thiofri_04469	Thiowin_04859, Thiowin_04888	Thiosp_00860, Thiosp_04410
