# Supplementary material for: Thiorhodovibrio frisius and Trv. litoralis spp. nov., Two Novel Members from a Clade of Fastidious Purple Sulfur Bacteria That Exhibit Unique Red-Shifted Light-Harvesting Capabilities
Source: Microorganisms. 2023 Sep 25;11(10):2394. doi: 10.3390/microorganisms11102394 (PMC10609205; doi:10.3390/microorganisms11102394)
Supplement: Supplementary file 1 [file microorganisms-11-02394-s001.zip › Tables S7-S10_2023.09.05.pdf]

Table S7. Genes of *Trv. frisius* DSM111777^T, *Trv. winogradskyi* DSM 6702^T, *Trv. litoralis* 06511^T involved in sulfur metabolism annotated through KEGG, RAST, and manually. (1/3)

Gene	Description	KEGG code	*Trv. frisius* 006511^T	*Trv. winogradskyi* DSM 6702^T	*Trv. litoralis* stain 970^T
2Fe-2S iron-sulfur cluster	2Fe-2S iron-sulfur cluster binding domain protein	-	Thiofri_01465	Thiowin_02587	Thiosp_03924
ABC transporter	ABC transporter, substrate-binding protein, aliphatic sulfonates family	-	Thiofri_02470	-	-
aprM	adenylylsulfate reductase, putative membrane anchor	-	Thiofri_03809	Thiowin_00432	Thiosp_01612
aprB	adenylylsulfate reductase, subunit B	K00395	Thiofri_03808	Thiowin_00433	Thiosp_01613
aprA	adenylylsulfate reductase, subunit A	K00394	Thiofri_03807	Thiowin_00434	Thiosp_01614
Arylsulfatase	Arylsulfatase	-	Thiofri_02489	-	Thiosp_04764
atsA 1	Arylsulfatase	-	Thiofri_01662	Thiowin_01532	Thiosp_03518
atsA 2	Arylsulfatase	-	Thiofri_02024	Thiowin_04338	Thiosp_04768
atsA 3	Arylsulfatase precursor	-	Thiofri_02494	-	-
atsB	Anaerobic sulfatase-maturating enzyme	-	Thiofri_03176	-	-
bchL	ferredoxin:protochlorophyllide reductase, Fe-S ATP-binding protein	-	Thiofri_04154	Thiowin_00758	Thiosp_00715
bcr 1	Sulfonamide resistance protein	-	Thiofri_03590	Thiowin_00679	Thiosp_00849
bcr 2	Sulfonamide resistance protein	-	-	-	Thiosp_02057
chuR	Anaerobic sulfatase-maturating enzyme	-	Thiofri_01670	-	-
cysA 1	Sulfate/thiosulfate import ATP-binding protein	K02045	Thiofri_01536	Thiowin_04178	Thiosp_02421
cysA 2	Sulfate/thiosulfate import ATP-binding protein	-	Thiofri_02713	-	-
cysC	Adenylyl-sulfate kinase	-	-	Thiowin_00440	Thiosp_02505
cysE	serine O-acetyltransferase	K00640	Thiofri_04252	Thiowin_00261	Thiosp_00506
cysH 1	Phosphoadenosine phosphosulfate reductase	-	-	Thiowin_04141	Thiosp_02726
cysH 2	Phosphoadenosine phosphosulfate reductase	-	-	Thiowin_04146	-
ddhA	Dimethylsulfide dehydrogenase subunit alpha precursor	-	-	-	Thiosp_04187
disulfide bond B	disulfide bond formation protein B	-	-	Thiowin_01987	-
dsbA 1	Thiol:disulfide interchange protein precursor	-	Thiofri_00685	Thiowin_01988	Thiosp_01192
dsbA 2	Thiol:disulfide interchange protein precursor	-	Thiofri_03576	Thiowin_02628	Thiosp_04608
dsbB	Disulfide oxidoreductase	-	Thiofri_00683	-	Thiosp_04609
dsbC	Thiol:disulfide interchange protein precursor	-	Thiofri_02780	Thiowin_01861	Thiosp_02390
dsbD 1	Thiol:disulfide interchange protein precursor	-	Thiofri_01064	Thiowin_00530	Thiosp_00809
dsbD 2	Thiol:disulfide interchange protein precursor	-	Thiofri_03598	Thiowin_01631	Thiosp_01176
dsrA	dissimilatory sulfite reductase alpha subunit	K11180	Thiofri_03979	Thiowin_00378	Thiosp_04058
dsrB	dissimilatory sulfite reductase beta subunit	K11181	Thiofri_03978	Thiowin_00379	Thiosp_04057
dsrC	Intracellular sulfur oxidation protein	-	Thiofri_03977	Thiowin_00380	Thiosp_04056
dsrE	Putative sulfurtransferase	-	Thiofri_03977	Thiowin_00380	Thiosp_04056
dsrF	Intracellular sulfur oxidation protein	-	Thiofri_03976	Thiowin_00381	Thiosp_04055
dsrH	Intracellular sulfur oxidation protein	-	Thiofri_03974	Thiowin_00383	Thiosp_04053
dsrM	Intracellular sulfur oxidation protein	-	Thiofri_03972	Thiowin_00385	Thiosp_04051
dsrK	Intracellular sulfur oxidation protein	-	Thiofri_03971	Thiowin_00386	Thiosp_04050
dsrL	Intracellular sulfur oxidation protein	-	Thiofri_03970	Thiowin_00387	Thiosp_04049
dsrJ	Intracellular sulfur oxidation protein	-	Thiofri_03969	Thiowin_00388	Thiosp_04048
dsrO	Intracellular sulfur oxidation protein	-	Thiofri_03968	Thiowin_00389	Thiosp_04047
dsrP	Intracellular sulfur oxidation protein	-	Thiofri_03967	Thiowin_00390	Thiosp_04046
dsrN	Intracellular sulfur oxidation protein	-	Thiofri_03966	Thiowin_00391	Thiosp_04045
dsrR	Intracellular sulfur oxidation protein	-	Thiofri_03965	Thiowin_00392	Thiosp_04044
dsrS	Intracellular sulfur oxidation protein	-	Thiofri_03964	Thiowin_00393	Thiosp_04043
erpA 1	Iron-sulfur cluster insertion protein ErpA	-	Thiofri_01419	Thiowin_00786	Thiosp_01240
erpA 2	Iron-sulfur cluster insertion protein ErpA	-	Thiofri_01422	Thiowin_00789	Thiosp_01243
fccB 1	Sulfide dehydrogenase [flavocytochrome c] flavoprotein chain precursor	K17229	Thiofri_00955	Thiowin_03811	Thiosp_00360
fccB 2	Sulfide dehydrogenase [flavocytochrome c] flavoprotein chain precursor	K17229	Thiofri_04639	Thiowin_04304	Thiosp_00839
fccB 3	Sulfide dehydrogenase [flavocytochrome c] flavoprotein chain precursor	K17229	-	Thiowin_04954	Thiosp_01680
fccA	cytochrome subunit of sulfide dehydrogenase	K17230	Thiofri_04640	Thiowin_03812<br>Thiowin_04953	Thiosp_00359<br>Thiosp_00840<br>Thiosp_01681
Formylglycine-generating sulfatase enzyme	Formylglycine-generating sulfatase enzyme	-	Thiofri_01195	-	-
frdB	Fumarate reductase iron-sulfur subunit	-	Thiofri_02433	Thiowin_02199	Thiosp_02570
glpE 1	Thiosulfate sulfurtransferase	-	-	Thiowin_01009	Thiosp_00285
glpE 2	Thiosulfate sulfurtransferase	-	-	Thiowin_04777	Thiosp_03227
Glycolipid sulfotransferase 1	Glycolipid sulfotransferase	-	Thiofri_03329	Thiowin_01553	Thiosp_03079
Glycolipid sulfotransferase 2	Glycolipid sulfotransferase	-	Thiofri_02084	-	-
Glycolipid sulfotransferase 3	Glycolipid sulfotransferase	-	Thiofri_03154	-	-
Glycolipid sulfotransferase 4	Glycolipid sulfotransferase	-	Thiofri_03431	-	-
hip	High-potential iron-sulfur protein precursor	-	Thiofri_01507	Thiowin_03612	Thiosp_02354

Table S7. (continued 2/3)

Gene	Description	KEGG code	*Trv. frisius* 006511^T	*Trv. winogradskyi* DSM 6702^T	*Trv. litoralis* stain 970^T
Fe-S cluster repair di-iron protein	iron-sulfur cluster repair di-iron protein	-	-	-	Thiosp_01675
iscA	Iron-sulfur cluster assembly protein	-	Thiofri_04618	Thiowin_05010	Thiosp_00251
metA	homoserine O-succinyltransferase/O-acetyltransferase	K00651	Thiofri_03616	Thiowin_04204	Thiosp_01598
metZ	O-succinylhomoserine sulfhydrylase	K10764	Thiofri_02417	Thiowin_01920	Thiosp_02563
moaD	Sulfur carrier protein	-	Thiofri_02058	Thiowin_03805	Thiosp_02808
moeZ 1	putative adenylyltransferase/sulfurtransferase	-	Thiofri_01145	Thiowin_03616	Thiosp_01781
moeZ 2	putative adenylyltransferase/sulfurtransferase	-	Thiofri_01505	Thiowin_03952	Thiosp_01810
moeZ 3	putative adenylyltransferase/sulfurtransferase	-	Thiofri_01904	Thiowin_04257	Thiosp_02523
moeZ 4	putative adenylyltransferase/sulfurtransferase	-	Thiofri_03751	-	Thiosp_03134
moeZ 5	putative adenylyltransferase/sulfurtransferase	-	-	-	Thiosp_03275
msrA 1	Peptide methionine sulfoxide reductase	-	Thiofri_04119	Thiowin_04189	Thiosp_00058
msrA 2	Peptide methionine sulfoxide reductase	-	Thiofri_04910	Thiowin_04645	Thiosp_01071
msrB	Peptide methionine sulfoxide reductase	-	Thiofri_02674	Thiowin_01774	Thiosp_03006
nifS	Cysteine desulfurase	-	Thiofri_03253	Thiowin_02439	-
petA	Ubiquinol-cytochrome c reductase iron-sulfur subunit	-	Thiofri_00562	Thiowin_01122	Thiosp_00461
phsC	thiosulfate reductase cytochrome b subunit	K08354	Thiofri_01459	Thiowin_02593	Thiosp_03930
pspE	Thiosulfate sulfurtransferase PspE precursor	-	Thiofri_04596	-	-
psrA	Sulfur reductase chain A	-	Thiofri_00885	-	-
isomerase 1	Protein-disulfide isomerase	-	-	-	Thiosp_02174
isomerase 2	Protein-disulfide isomerase	-	-	-	Thiosp_03113
putative iron-sulfur cluster-binding protein	putative iron-sulfur cluster-binding protein	-	-	-	Thiosp_01691
cysZ	putative sulfate transport protein	-	Thiofri_02456	-	Thiosp_00022
putative sulfate transporter	putative sulfate transporter	-	Thiofri_04214	Thiowin_04056	Thiosp_00922
putative sulfate transporter	putative sulfate transporter	-	Thiofri_02282	Thiowin_03509	Thiosp_03919
putative sulfate transporter	putative sulfate transporter	-	-	-	Thiosp_04749
dndC	putative sulfurtransferase	-	-	-	Thiosp_02750
sqrD	sulfide:quinone oxidoreductase	K17218	Thiofri_01966	Thiowin_01274	Thiosp_02348
sqrF	sulfide:quinone oxidoreductase	K17218	Thiofri_02206	Thiowin_02851	Thiosp_01541
soeA	sulfite dehydrogenase (quinone) subunit A	K21307	Thiofri_03816	Thiowin_04202	Thiosp_01600
soeB	sulfite dehydrogenase (quinone) subunit B	K21308	Thiofri_03815	Thiowin_04201	Thiosp_01601
soeC	sulfite dehydrogenase (quinone) subunit C	K21309	Thiofri_03814	Thiowin_04200	Thiosp_01602
soxA	L-cysteine S-thiosulfotransferase	K17222	-	-	Thiosp_02372
soxX	L-cysteine S-thiosulfotransferase	K17223	-	-	Thiosp_02371
soxB	S-sulfosulfanyl-L-cysteine sulfohydrolase	K17224	-	-	Thiosp_02368
SoxY	sulfur oxidation protein	K17226	Thiofri_04248	Thiowin_00257	Thiosp_00502
soxZ	sulfur-oxidizing protein	K17227	Thiofri_04249	Thiowin_00258	Thiosp_00503
redox-active disulfide protein 2	redox-active disulfide protein 2	-	Thiofri_01884	Thiowin_00948	Thiosp_04292
redox-active disulfide protein 2	redox-active disulfide protein 2	-	Thiofri_03863	Thiowin_02022	-
resA	Thiol-disulfide oxidoreductase	-	Thiofri_01233	Thiowin_02240	Thiosp_01220
rhdA	Thiosulfate sulfurtransferase	-	-	Thiowin_01548	-
sat	Sulfate adenylyltransferase	K00958	Thiofri_03810	Thiowin_00431	Thiosp_01611
sdhC	Succinate dehydrogenase	-	Thiofri_03070	Thiowin_01091	Thiosp_03026
sdhD	Succinate dehydrogenase	-	Thiofri_03071	Thiowin_01092	Thiosp_03027
sdhA	Succinate dehydrogenase	-	Thiofri_03072	Thiowin_01093	Thiosp_03028
sdhB	Succinate dehydrogenase iron-sulfur subunit	-	Thiofri_03073	Thiowin_01094	Thiosp_03029
sgpA 1	Sulfur globule protein CV1 precursor	-	Thiofri_01608	Thiowin_00268	-
sgpA 2	Sulfur globule protein CV1 precursor	-	Thiofri_04295	Thiowin_02802	-
sseA	3-mercaptopyruvate sulfurtransferase	-	Thiofri_01914	-	-
iron-sulfur subunit reductase 1	succinate dehydrogenase/fumarate reductase iron-sulfur subunit	-	Thiofri_03972	Thiowin_00540	Thiosp_01888
iron-sulfur subunit reductase 2	succinate dehydrogenase/fumarate reductase iron-sulfur subunit	-	Thiofri_02925	Thiowin_03147	
sufE 1	Cysteine desulfuration protein	-	Thiofri_00626	Thiowin_00610	Thiosp_00250
sufE 2	Cysteine desulfuration protein	-	Thiofri_04617	Thiowin_05011	Thiosp_04458
Sulfite exporter TauE/SafE	Sulfite exporter	-	Thiofri_02039	Thiowin_03913	Thiosp_01779
Sulfotransferase domain protein	Sulfotransferase domain protein	-	Thiofri_00084	Thiowin_00890	Thiosp_00745
Sulfotransferase domain protein	Sulfotransferase domain protein	-	Thiofri_03647	-	Thiosp_01275
Sulfotransferase domain protein	Sulfotransferase domain protein	-	Thiofri_03635	-	Thiosp_01307
Sulfotransferase domain protein	Sulfotransferase domain protein	-	Thiofri_00082	-	-
Sulfotransferase domain protein	Sulfotransferase domain protein	-	Thiofri_03646	-	-
Sulfotransferase family protein	Sulfotransferase family protein	-	Thiofri_01058	Thiowin_04831	Thiosp_00805
Sulfotransferase family protein	Sulfotransferase family protein	-	Thiofri_04087	-	-
Sulfur oxygenase reductase	Sulfur oxygenase reductase	-	-		Thiosp_00861
TusD/DsrE 1	sulfur relay protein	-	-	Thiowin_02441	-
TusD/DsrE 2	sulfur relay protein	-	-	Thiowin_02484	-
TusE/DsrC/DsvC	sulfur relay protein, TusE/DsrC/DsvC family	-	-	Thiowin_03470	-
thiF	Sulfur carrier protein ThiS adenylyltransferase	-	Thiofri_03134	Thiowin_01051	-

Table S7. (continued 3/3)

Gene	Description	KEGG code	*Trv. frisius* 006511^T	*Trv. winogradskyi* DSM 6702^T	*Trv. litoralis* stain 970^T
thiol-disulfide oxidoreductase	thiol-disulfide oxidoreductase	-	Thiofri_04208	Thiowin_04049	Thiosp_00810
thiol-disulfide oxidoreductase	thiol-disulfide oxidoreductase	-	-	-	Thiosp_00929
thiosulfate reductase cytochrome B subunit	thiosulfate reductase cytochrome B subunit	-	Thiofri_01577	Thiowin_02836	Thiosp_02858
thiosulfate reductase cytochrome B subunit	thiosulfate reductase cytochrome B subunit	-	Thiofri_01459	Thiowin_02593	Thiosp_03930
thiosulfate sulfurtransferase	thiosulfate sulfurtransferase	-	Thiofri_01783	Thiowin_02412	Thiosp_03786
thiosulfate sulfurtransferase	thiosulfate sulfurtransferase	-	-	-	Thiosp_04325
ttrB	tetrathionate reductase subunit B	K08358	Thiofri_03969	Thiowin_00388	Thiosp_04048
tusA	Sulfurtransferase	-	Thiofri_03750	Thiowin_02127	Thiosp_03274
tusE 1	Sulfurtransferase	-	Thiofri_02930	Thiowin_00374	Thiosp_00391
tusE 2	Sulfurtransferase	-	Thiofri_03019	Thiowin_00383	Thiosp_01069
tusE 3	Sulfurtransferase	-	Thiofri_03974	Thiowin_01425	Thiosp_01672
tusE 4	Sulfurtransferase	-	Thiofri_03983	Thiowin_01970	Thiosp_03114
tusE 5	Sulfurtransferase	-	-	Thiowin_01971	Thiosp_03194
tusE 6	Sulfurtransferase	-	-	Thiowin_01990	Thiosp_03228
tusE 7	Sulfurtransferase	-	-	Thiowin_03145	Thiosp_03279
tusE 8	Sulfurtransferase	-	-	Thiowin_03310	Thiosp_03380
tusE 9	Sulfurtransferase	-	-	Thiowin_03810	Thiosp_04053
tusE 10	Sulfurtransferase	-	-	-	Thiosp_04063
ydeM 1	Anaerobic sulfatase-maturating enzyme	-	-	Thiowin_01440	Thiosp_03823
ydeM 2	Anaerobic sulfatase-maturating enzyme	-	-	-	Thiosp_04706
ydeM 3	Anaerobic sulfatase-maturating enzyme	-	-	-	Thiosp_04736

Table S8. Hydrogenases related genes of *Thiorhodovibrio spp*.

Gene	Description	***Trv. frisius*** **006511^T**	***Trv. winogradskyi*** **DSM 6702^T**	***Trv. litoralis*** **stain 970^T**
Coenzyme F420b	Reducing hydrogenase, subunit beta	Thiofri_01984	Thiowin_01243	Thiosp_02229
Coenzyme F420a	Reducing hydrogenase, alpha subunit	Thiofri_02317	Thiowin_01546	Thiosp_02302
hndA	NADP-reducing hydrogenase subunit HndA	Thiofri_01447	Thiowin_02604	Thiosp_03940
hndC	NADP-reducing hydrogenase subunit HndC	Thiofri_01446	Thiowin_02605	Thiosp_03941
hndD	NADP-reducing hydrogenase subunit HndC	Thiofri_01445	Thiowin_02607	Thiosp_03943
hoxG	Uptake hydrogenase large subunit	-	Thiowin_04278	Thiosp_04182
hoxH	NAD-reducing hydrogenase HoxS subunit beta	Thiofri_01443	Thiowin_02609	Thiosp_03945
hoxK 1	Uptake hydrogenase small subunit precursor	Thiofri_00584	Thiowin_00538	Thiosp_01890
hoxK 2	Uptake hydrogenase small subunit precursor	Thiofri_02923	Thiowin_03149	Thiosp_04181
hoxK 3	Uptake hydrogenase small subunit precursor	-	Thiowin_04277	-
hoxY	NAD-reducing hydrogenase HoxS subunit delta	Thiofri_01444	Thiowin_02608	Thiosp_03944
hupC 1	Putative Ni/Fe-hydrogenase B-type cytochrome subunit	Thiofri_00586	Thiowin_04279	Thiosp_04183
hupC 2	Putative Ni/Fe-hydrogenase B-type cytochrome subunit	Thiofri_00605	Thiowin_04325	Thiosp_04453
hyaB 1	Hydrogenase-1 large chain	Thiofri_00585	Thiowin_00541	Thiosp_01887
hyaB 2	Hydrogenase-1 large chain	Thiofri_02926	Thiowin_03146	Thiosp_02759
hyaC	Putative Ni/Fe-hydrogenase 1 B-type cytochrome subunit	Thiofri_02696	Thiowin_03157	Thiosp_02350
hybB	Putative Ni/Fe-hydrogenase 2 b-type cytochrome subunit	Thiofri_01455	Thiowin_02597	Thiosp_03934
hybC 1	Hydrogenase-2 large chain precursor	Thiofri_01454	Thiowin_02598	Thiosp_03935
hybC 2	Hydrogenase-2 large chain precursor	-	-	Thiosp_04191
hybD 1	Hydrogenase 2 maturation protease	Thiofri_00587	Thiowin_02599	Thiosp_03936
hybD 2	Hydrogenase 2 maturation protease	Thiofri_00608	Thiowin_04280	Thiosp_04184
hybD 3	Hydrogenase 2 maturation protease	Thiofri_01221	-	-
hybD 4	Hydrogenase 2 maturation protease	Thiofri_01453	-	-
hybO	Hydrogenase-2 small chain precursor	Thiofri_01457	Thiowin_02595	Thiosp_03932
hydrogenase 3	Hydrogenase 3 maturation protease	Thiofri_01442	Thiowin_02610	Thiosp_03946
hydrogenase	Hydrogenase assembly chaperone	Thiofri_04418	Thiowin_00323	-
hydrogenase	Hydrogenase nickel incorporation protein	Thiofri_04882	-	Thiosp_00099
hyfB 1	Hydrogenase-4 component B	Thiofri_04365	Thiowin_00048	Thiosp_00423
hyfB 2	Hydrogenase-4 component B	Thiofri_04366	Thiowin_01315	Thiosp_00424
hyfB 3	Hydrogenase-4 component B	Thiofri_04367	Thiowin_01316	Thiosp_00425
hyfB 4	Hydrogenase-4 component B	-	Thiowin_01317	-
hynB1	Periplasmic [NiFe] hydrogenase small subunit 1 precursor	-	-	Thiosp_02758
hypA	Hydrogenase/urease nickel incorporation	-	Thiowin_04705	-
hypB	Hydrogenase isoenzymes nickel incorporation	Thiofri_04923	Thiowin_04659	Thiosp_00072
hypC 1	Hydrogenase isoenzymes formation protein	Thiofri_02316	Thiowin_01241	Thiosp_03280
hypC 2	Hydrogenase isoenzymes formation protein	-	-	Thiosp_04364
hypD	Hydrogenase expression/formation protein	Thiofri_02315	Thiowin_01240	Thiosp_03281
hypE	Hydrogenase expression/formation protein	Thiofri_02314	Thiowin_01239	Thiosp_03282
Ni/Fe-hydrogenase	*b-type cytochrome subunit*	-	Thiowin_02554	-
Soluble hydrogenase	*42 kDa subunit*	Thiofri_03672	-	-

Table S9. Genes of *Trv. frisius* DSM111777^T, *Trv. winogradskyi* DSM 6702^T, *Trv. litoralis* 06511^T involved in carbon metabolism annotated through KEGG online database.

Gene	Description	KO code	*Trv. frisius 006511^T*	*Trv. winogradskyi* DSM 6702^T	*Trv. litoralis* stain 970^T
IDH1, IDH2, icd	isocitrate dehydrogenase	K00031	Thiofri_01855	Thiowin_01731	Thiosp_03395
sdhA, frdA	succinate dehydrogenase flavoprotein subunit	K00239	Thiofri_02434, Thiofri_03072	Thiowin_01093, Thiowin_02200	Thiosp_02571, Thiosp_03028
sdhB, frdB	succinate dehydrogenase iron-sulfur subunit	K00240	Thiofri_02433, Thiofri_03073	Thiowin_01094	Thiosp_02570, Thiosp_03029
sdhC, frdC	succinate dehydrogenase cytochrome b subunit	K00241	Thiofri_03070	Thiowin_01091	Thiosp_03026
sdhD, frdD	succinate dehydrogenase membrane anchor subunit	K00242	Thiofri_03071	Thiowin_01092	Thiosp_03027
metF, MTHFR	methylenetetrahydrofolate reductase (NADH)	K00297	Thiofri_04212	Thiowin_04055	Thiosp_00923
ackA	acetate kinase	K00925	Thiofri_02256	Thiowin_03935	Thiosp_03554
ppdK	pyruvate, orthophosphate dikinase	K01006	Thiofri_02310	Thiowin_01234	Thiosp_03286
pps, ppsA	pyruvate, water dikinase	K01007	Thiofri_04696	Thiowin_03492, Thiowin_04722	Thiosp_04666
folD	methylenetetrahydrofolate dehydrogenase (NADP+)	K01491	Thiofri_02694	Thiowin_01484	Thiosp_02352
ppc	phosphoenolpyruvate carboxylase	K01595	Thiofri_01788	Thiowin_02645	Thiosp_01620
fumA, fumB	fumarate hydratase, class I	K01676	Thiofri_01973	Thiowin_01540	Thiosp_02230
ACO, acnA	aconitate hydratase	K01681	Thiofri_04001	Thiowin_04240	Thiosp_01407
acs	acetyl-CoA synthetase	K01895	Thiofri_03923	Thiowin_02135	Thiosp_03629
sucD	succinyl-CoA synthetase alpha subunit	K01902	Thiofri_04775	Thiowin_05000	Thiosp_00129
sucC	succinyl-CoA synthetase beta subunit	K01903	Thiofri_04776	Thiowin_05001	Thiosp_00130
accC	acetyl-CoA carboxylase, biotin carboxylase subunit	K01961	Thiofri_03602	Thiowin_01627	Thiosp_01172
accA	acetyl-CoA carboxylase carboxyl transferase subunit alpha	K01962	Thiofri_01789	Thiowin_02644	Thiosp_01621
accD	acetyl-CoA carboxylase carboxyl transferase subunit beta	K01963	Thiofri_02412	Thiowin_01915	Thiosp_02558
accB, bccP	acetyl-CoA carboxylase biotin carboxyl carrier protein	K02160	Thiofri_03601	Thiowin_01628	Thiosp_01173
por, nifJ	pyruvate-ferredoxin/flavodoxin oxidoreductase	K03737	Thiofri_01572	Thiowin_02842	Thiosp_01649
pta	phosphate acetyltransferase	K13788	Thiofri_02257	Thiowin_03934	Thiosp_03553
maeB	malate dehydrogenase	K00029	Thiofri_04337	Thiowin_00275	Thiosp_00363
GAPDH, gapA	glyceraldehyde 3-phosphate dehydrogenase	K00134	Thiofri_01882, Thiofri_03859, Thiofri_04206	Thiowin_02026, Thiowin_04047	Thiosp_00075, Thiosp_00931
tktA, tktB	transketolase	K00615	Thiofri_04207	Thiowin_01514, Thiowin_03464, Thiowin_04048	Thiosp_00930, Thiosp_03193
prkB	phosphoribulokinase	K00855	Thiofri_00011	Thiowin_00010	Thiosp_00011
pgk	phosphoglycerate kinase	K00927	Thiofri_04204	Thiowin_04046	Thiosp_00932
ppdK	pyruvate, orthophosphate dikinase	K01006	Thiofri_02310	Thiowin_01234	Thiosp_03286
ppc	phosphoenolpyruvate carboxylase	K01595	Thiofri_01788	Thiowin_02645	Thiosp_01620
rbcL, cbbL	ribulose-bisphosphate carboxylase large chain	K01601	Thiofri_00013, Thiofri_03064, Thiofri_04982	Thiowin_00012, Thiowin_01572, Thiowin_01759, Thiowin_04628	Thiosp_00041, Thiosp_00266, Thiosp_03020
rbcS, cbbS	ribulose-bisphosphate carboxylase small chain	K01602	Thiofri_03063	Thiowin_01760	Thiosp_03019
xfp, xpk	xylulose-5-phosphate/fructose-6-phosphate phosphoketolase	K01621	Thiofri_01636, Thiofri_02580	Thiowin_01664, Thiowin_03240	Thiosp_01912
fbaA	fructose-bisphosphate aldolase, class II	K01624	Thiofri_04202	Thiowin_04044	Thiosp_00934
rpe	ribulose-phosphate 3-epimerase	K01783	Thiofri_03927	Thiowin_03832	Thiosp_03736
tpiA	triosephosphate isomerase (TIM)	K01803	Thiofri_03015	Thiowin_01864	Thiosp_03325
rpiA	ribose 5-phosphate isomerase A	K01807	Thiofri_04351	Thiowin_01330	Thiosp_00410
fbp	fructose-1,6-bisphosphatase I	K03841	Thiofri_03755	Thiowin_04261	Thiosp_01574

Table S10. Genes of *Trv. frisius* DSM 111777^T, *Trv. winogradskyi* DSM 6702^T, *Trv. litoralis* 06511^T involved in nitrogen metabolism annotated through KEGG online database and Geneious.

Gene	Description	KEGG code	*Trv. frisius 006511^T*	*Trv. winogradskyi* DSM 6702^T	*Trv. litoralis* stain 970^T
Hydrolase	ADP-ribosyl-[dinitrogen reductase] hydrolase	-	-	-	Thiosp_02089
can	carbonic anhydrase	K01673	Thiofri_02566, Thiofri_02875	-	Thiosp_04803
cah	carbonic anhydrase	K01674	-	-	Thiosp_04665
cowN	CO weal-nitrogenase	-	Thiofri_01782	Thiowin_02413	Thiosp_01780
Dinitrogenase cofactor 1	Dinitrogenase iron-molybdenum cofactor	-	Thiofri_04121	Thiowin_04191	Thiosp_01060
Dinitrogenase cofactor 2	Dinitrogenase iron-molybdenum cofactor	-	Thiofri_03621	Thiowin_04210	Thiosp_01592
draG 1	ADP-ribosyl-[dinitrogen reductase] glycohydrolase	-	Thiofri_01467	Thiowin_02508	Thiosp_03922
draG 2	ADP-ribosyl-[dinitrogen reductase] glycohydrolase	-	Thiofri_03311	Thiowin_02585	Thiosp_00980
draT	NAD(+)--dinitrogen-reductase ADP-D-ribosyltransferase	-	Thiofri_01194	Thiowin_00052	-
glnA	glutamine synthetase	K01915	Thiofri_04606	Thiowin_04786	Thiosp_00295
glnB 1	Nitrogen regulatory protein P-II	-	Thiofri_01494	Thiowin_01382	Thiosp_00128
glnB 2	Nitrogen regulatory protein P-II	-	Thiofri_04774	Thiowin_04999	Thiosp_02763
glnG 1	Nitrogen regulation protein NR(I)	-	Thiofri_04602	Thiowin_00022	Thiosp_00292
glnG 2	Nitrogen regulation protein NR(I)	-	-	Thiowin_04783	Thiosp_04410
glnL	Nitrogen regulation protein NR(II)	-	Thiofri_04603	Thiowin_04784	Thiosp_00293
gltB	glutamate synthase (NADPH) large chain	K00265	Thiofri_04701	Thiowin_04758	Thiosp_04700
gltD	glutamate synthase (NADPH) small chain	K00266	Thiofri_04700	Thiowin_04759	Thiosp_04701
hcp	hydroxylamine reductase	K05601	Thiofri_03474	Thiowin_04302	Thiosp_01683, Thiosp_02885, Thiosp_03355
narI	nitrate reductase gamma subunit	K00374	Thiofri_03973	Thiowin_00384	Thiosp_04052
NtcA	global nitrogen regulator	-	-	Thiowin_03210	-
nifD 1	Nitrogenase molybdenum-iron protein alpha chain	K02586	Thiofri_01039	Thiowin_00876	Thiosp_00781
nifD 2	Nitrogenase molybdenum-iron protein alpha chain	K02586	Thiofri_01329	Thiowin_04854	Thiosp_03837
nifH1 2	Nitrogenase iron protein 1	K02588	Thiofri_01038	Thiowin_04855	Thiosp_00780
nifK 1	Nitrogenase molybdenum-iron protein beta chain	K02591	Thiofri_01040	Thiowin_00875	Thiosp_00782
nifK 2	Nitrogenase molybdenum-iron protein beta chain	K02591	-	Thiowin_04853	Thiosp_03836
nifL 1	Nitrogen fixation regulatory protein	-	Thiofri_00864	Thiowin_00699	Thiosp_03825
nifL 2	Nitrogen fixation regulatory protein	-	Thiofri_01299	Thiowin_00954	Thiosp_04256
nifL 3	Nitrogen fixation regulatory protein	-	Thiofri_01450	Thiowin_02314	Thiosp_04626
nifL 4	Nitrogen fixation regulatory protein	-	Thiofri_04381	Thiowin_02958	-
nifW	Nitrogenase-stabilizing/protective protein	-	Thiofri_02033	Thiowin_03920	Thiosp_03511
nifX 1	nitrogen fixation protein	-	Thiofri_01043	Thiowin_04850	Thiosp_00785
nifX 2	nitrogen fixation protein	-	Thiofri_01330	Thiowin_00874	Thiosp_03835
P-II	Nitrogen regulatory protein P-II	-	-	-	Thiosp_02267
nitrogenase	nitrogenase-associated protein	-	Thiofri_01530	-	Thiosp_01215
nreC 1	Nitrogen regulation protein C	-	-	Thiowin_01945	Thiosp_02664
nreC 2	Nitrogen regulation protein C	-	-	Thiowin_03114	Thiosp_04744
ntrC	Nitrogen assimilation regulatory protein	-	Thiofri_02851	Thiowin_01825	Thiosp_03613
ptsN	Nitrogen regulatory protein	-	Thiofri_04725	Thiowin_00316	Thiosp_04352
N2 fixation	putative nitrogen fixation protein	-	Thiofri_01332	Thiowin_00872	Thiosp_03833
FixT	putative nitrogen fixation protein	-	Thiofri_01041	Thiowin_04852	Thiosp_00783
rnfB 1	Nitrogen fixation protein	-	Thiofri_00862	Thiowin_00956	Thiosp_03600
rnfB 2	Nitrogen fixation protein	-	Thiofri_02816	Thiowin_03587	Thiosp_04258
rnfC 1	Nitrogen fixation protein	-	Thiofri_00861	Thiowin_00957	Thiosp_03599
rnfC 2	Nitrogen fixation protein	-	Thiofri_02815	Thiowin_03586	Thiosp_04259
rnfD 1	Nitrogen fixation protein	-	Thiofri_00860	Thiowin_00958	Thiosp_03598
rnfD 2	Nitrogen fixation protein	-	Thiofri_02814	Thiowin_03585	Thiosp_04260
rnfE 1	Nitrogen fixation protein	-	Thiofri_00858	Thiowin_00960	Thiosp_03596
rnfE 2	Nitrogen fixation protein	-	Thiofri_02812	Thiowin_03583	Thiosp_04262
rnfG	Nitrogen fixation protein	-	Thiofri_00859	Thiowin_00959	Thiosp_03597
rnfG 2	Nitrogen fixation protein	-	Thiofri_02813	Thiowin_03584	Thiosp_04261
Signal-histidine kinase	Signal transduction histidine kinase, nitrogen specific	-	Thiofri_04893	-	-
